# Emergency ward ultrasound: clinical audit on disinfection practices during routine and sterile examinations

**DOI:** 10.1186/s13756-021-00896-w

**Published:** 2021-01-30

**Authors:** A. Andolfo, R. Maatoug, N. Peiffer-Smadja, C. Fayolle, K. Blanckaert

**Affiliations:** 1 Service d’accueil des urgences/Structure mobile d’urgence et de reanimation, Centre Hospitalier de Dunkerque, 59140 Dunkirk, France; 2 AP-HP, Service de Psychiatrie Adulte de la Pitié-Salpêtrière, Institut du Cerveau, ICM, Sorbonne Université, 75013 Paris, France; 3 French Institute for Medical Research (Inserm), Infection Antimicrobials Modelling Evolution (IAME), UMR 1137, University Paris Diderot, Paris, France; 4 National Institute for Health Research Health Protection Research Unit in Healthcare. Associated Infections and Antimicrobial Resistance, Imperial College London, London, UK; 5 Service de Réanimation et Unité de Soins Continus, Centre Hospitalier de Dunkerque, 59140 Dunkirk, France; 6 Centre d’appui et de prevention des infections associees aux soins (CPIAS) Pays de la Loire, Centre Hospitalier Universitaire de Nantes, 44093 Nantes, France

**Keywords:** Ultrasound probes, Clinical audit, Disinfection, Reusable medical devices, Cross-transmission

## Abstract

**Context:**

In the emergency ward, where the use of ultrasound is common (including for sterile procedures), ward equipment is constantly exposed to high risks of microbiological contamination. There are no clear guidelines for disinfection control practices in emergency departments, and it is not known how emergency ward doctors follow good hygiene practices.

**Method:**

A multi-centre audit was conducted in 16 emergency services from Northern France regional hospitals, in form of a questionnaire. It was proposed to all emergency ward physicians. We excluded questionnaires when physicians mentioned that they did not use ultrasound on a daily basis. The questionnaire was designed using existing hygiene and ultrasound disinfection practices guidelines from varying French medical societies. It included three different clinical scenarios: (a) ultrasound on healthy skin, (b) on injured skin, and (c) ultrasound-guided punctures. All questions were closed-ended, with only one answer corresponding to the guidelines. We then calculated compliance rates for each question, each clinical situation, and an overall compliance rate for all the questions.

**Results:**

104 questionnaires were collected, and 19 were excluded. For the 85 analysed questionnaires, the compliance rates were 60.4% 95% CI [56.4–64.7] for ultrasound on healthy skin, 70.9% 95% CI [66.3–76.1] on injured skin and 69.4% 95% CI [65.1–73.6] for ultrasound-guided punctures. The overall compliance rate for the compliance questions was 66.1% 95% CI [62.8–69.1]. Analysis of the questionnaires revealed severe asepsis errors, misuse of gel, ignorance of infection control practices to be applied in the context of ultrasound-guided puncture and exposure of the probe to body fluids.

**Conclusion:**

This study details areas for quality improvement in the disinfection of emergency ultrasound scanner use. Consequently, we propose a standardized protocol based upon the recommendations used for the questionnaire drafting, with a visual focus on the low compliance points that have been revealed in this audit. This protocol has been distributed to all the medical emergency services audited and included in the emergency resident’s ultrasound learning program.

## Introduction

### Background

Ultrasound systems are reusable medical devices that are increasingly used in the emergency ward. Over the last decade, scientific societies have given emergency physicians the possibility to practice and conduct more and more different types of examinations on their own, including invasive procedures [1].

The ultrasound probe is usually classified as a non-critical device by Spaulding's historical classification, and it only requires low level disinfection after patient contact [2].

However, when the ultrasound is used for an invasive procedure (e.g. inserting peripheral and central vein catheters, cavity puncture), there is a risk of contact with biological fluids, directly or through the sterile sheath. Moreover, various parts of the device, especially the probes, the keyboard and the gel vials are constantly exposed to bacterial contamination [3–5].

Good procedural practice recommendations for probe disinfection are currently available for semi-critical devices such as endocavitary ultrasound probes (EUP). The examinations carried out using these devices involve contacts with a mucous membrane, and sometimes an invasive sampling (e.g. prostate biopsies). General recommendations concerning good disinfection practices between two examinations were issued by the French Public Health institution in 2008, [6] and have been reinforced in 2016 [7], in a context of growing healthcare-associated infections.

These guidelines recommend performing a visual examination of the sheath itself and of the compress used to wipe the probe, to search for a potential blood or body fluid stain. If contamination of the probe is observed, an intermediate-level disinfection by soaking in a disinfectant solution is recommended. This sheath verification protocol is identical to the recommendations published by French anaesthesia and radiology societies. [8, 9].

Several recent assessments of professional practices (APP) have shown mediocre compliance rates with EUP hygiene measures: in France, one APP carried out with healthcare workers using EUP highlighted an ignorance of intermediate level disinfection procedures [10]. On a European scale, a survey of similar practices conducted by the European Radiological Society (ERS) describes the current level of compliance as worrying [11]. 11% of the surveyed radiologists did not use probe sheaths or covers for endocavitary examinations, and 23% of them did not do so during invasive procedures. Following this survey, the European recommendations were changed:

“The category ‘semi-critical’ as detailed in the Spaulding classification has been omitted. The generally accepted recommendations for disinfection are similar to those for critical procedures, i.e., transducers require high level disinfection or sterilization. [12]”.

### Objectives

The aforementioned APP were conducted on ultrasound professional practitioners, such as radiologists and gynaecologists, and not on emergency physicians. In France, there are no specific guidelines for ultrasound-guided invasive procedures. To date, neither hygiene practices of emergency ward doctors using ultrasound, nor their knowledge of existing ultrasound disinfection guidelines have been assessed. Their level of awareness of cross-contamination risks also remains to be determined, especially since emergency wards have a high potential for environmental contamination.

An audit was therefore conducted with the emergency services of our region. The objectives were to assess hygiene practices surrounding the use of the ultrasound system in the emergency department. We wanted to identify areas for improvement and propose a protocol for disinfecting ultrasound equipment based on actual hygiene guidelines.

## Methods

### Study design

The audit consisted in a declarative, anonymous, and multi-centre survey. It was conducted in all *Hauts-de-France* region (Northern France) region hospitals that had an emergency department equipped with a functional ultrasound system. In total, 29 hospital centres (24 public hospitals and 5 private establishments) were contacted, and all emergency department chiefs were invited to take part in the study by phone call.

### Participants

Once a hospital was included in the study, a survey coordinator was chosen among the medical team of each emergency department. There was no condition to become survey coordinator.

The survey link was sent to all survey coordinators, who were asked to relay it to all the practitioners who worked in their department. Practitioners included emergency physicians, other specialists that worked during daytime or nightshift in the emergency room, and current residents. Along with the link, a note detailed that the data would be used anonymously for a multi-centre survey and that practitioners were free not to participate. The note also emphasised the importance of answering honestly.

The entire questionnaire was completed online. The survey URL remained active for a period of 10 weeks, between September 14th and December 1st. At the end of October, a reminder was sent by email to every coordinator, to help maximise participation rates.

### Variables

The different parts of the questionnaire were:

- *Q.2* The possibility for practitioners who did not use ultrasound in their daily routine to end the survey. Those practitioners were excluded from further analysis and the online questionnaire automatically ended.
- *Q.1, Q.3 and “Age”* Variables collected for bivariate analysis. (Q.1 from excluded questionnaires were not considered).
- *Q.4–Q.13* Medical device cleaning resources and professional clothing.
- *Q.14–Q.32* Guideline application in three clinical situations: (a) ultrasound on healthy skin, (b) on injured skin and (c) ultrasound-guided procedures. These were questions with correct answers and compliance rates. The third part [(c) ultrasound-guided procedures] was optional, to account for the fact that some participants were not used to such procedures. This part also included 4 questions on sheath verification protocol after an invasive procedure.
- *Q.33–Q.35* Self-evaluation.

### Measurement

Compliance questions related to gestures before, during and after an ultrasound examination: hand hygiene, probe wiping, keyboard wiping, correct use of sterile supplies like gel or covers, use of individual protection equipment and gloves, all in the right order. Every move is described in the guidelines, and many uncontrolled situations can lead to environment and/or patient contamination. To rigorously build this section of the questionnaire, we used the following sources:

1. Device disinfection recommendations [13], standard and contact precautions [14, 15], hand hygiene [16], all from the *Société Française d’Hygiène Hospitalière* (SF2H).
2. For ultrasound-guided procedures: EUP recommendations from the *Haut Conseil de Santé Publique* [7].
3. For the sheath/cover checking protocol: recommendations from anaesthesiology [8], obstetric and radiology [9] societies.

Every question in this section was closed-ended, with one expected answer out of 2 or 3 propositions.

We determined compliance rates for each of the three clinical situations and a global compliance rate including all questions in this section, with confidence intervals. The questionnaire is fully available in Additional file 1.

### Bias

Self-reports can be vulnerable to bias. However, given that the healthcare professionals surveyed in this study have received little information, evaluation, and lack clear guidelines on good ultrasound hygiene practices, we argue that their conscious need for such recommendations minimizes bias.

### Statistical methods

Bivariate analysis: we compared every answer to our initial data:

- Age (< 30 yo / 30–45 yo / > 45 yo).
- Trained or not in hospital hygiene (Q.1).
- Ultrasound diploma or not (Q.3).

To identify a potential link between behaviour and validated qualitative characteristics, we used Fisher’s exact test (when the sample size permitted it). The significance threshold was *p* < 0.05.

Missing data: Surveys whose compliance questions parts had five or more not answered questions were excluded from analysis.

Software used: ‘SAS’, version 9.4.

## Results

### Participants

Sixteen out of the 29 (55%) hospitals in the *Hauts-de-France* region (Northern France) accepted to participate in the study.

Only 8 (50%, n = 16), declared that they had a protocol for ultrasound equipment disinfection.

104 questionnaires were completed and analysed. This represents 33% of all staff from the included emergency departments.

### Descriptive data

**Fig. 1:**
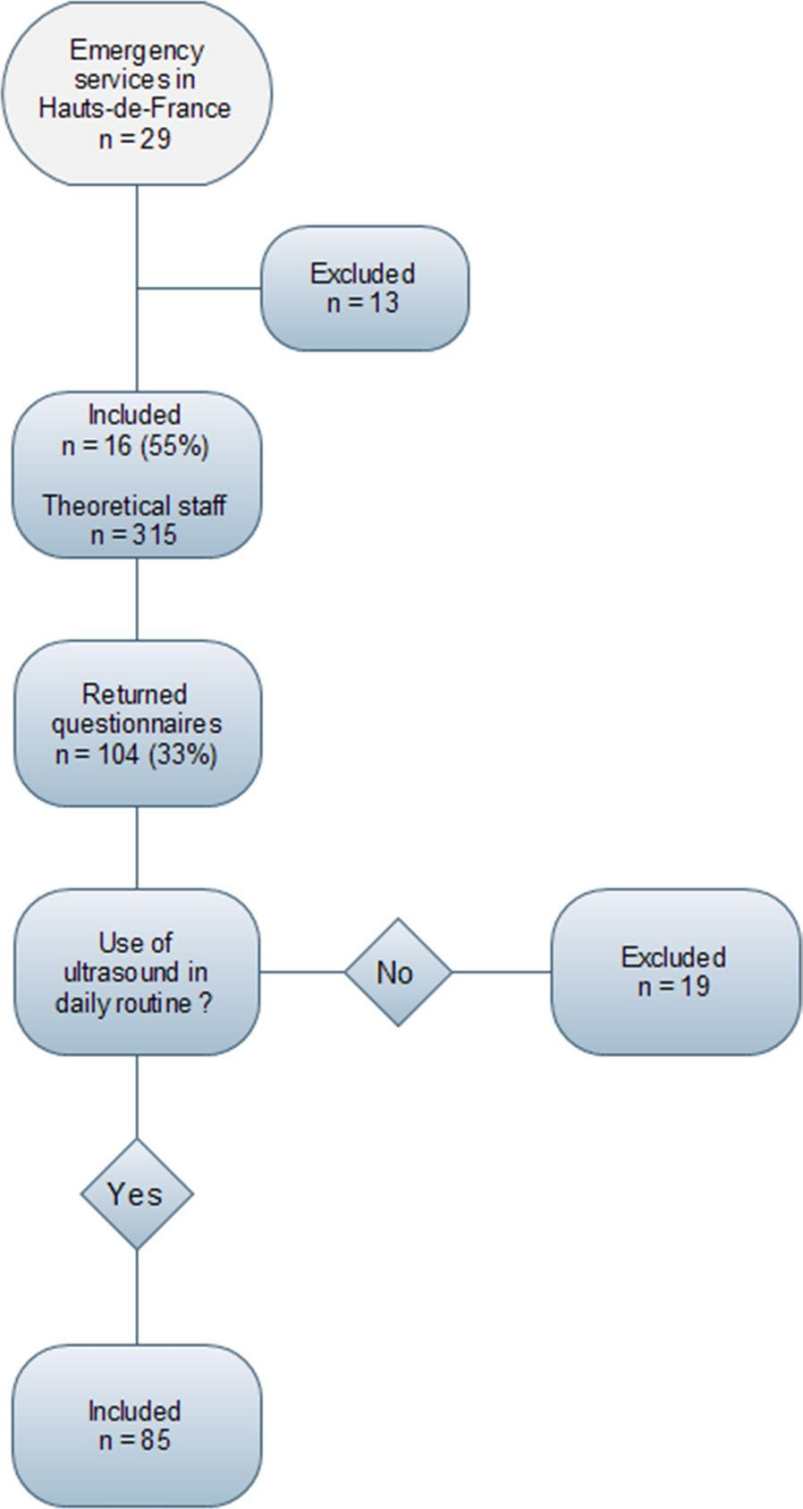
Surveys participation and inclusion flow-chart

Most of the participants (87%, n = 104) were hospital or attending physicians in emergency medicine and 58% (60, n = 104) of them were between 30 and 45 years old. 81 were trained (78%, n = 104) in hospital hygiene (local training by hygiene teams). 19 professionals (18%, n = 104) declared that they did not use ultrasound in their daily practice and stopped the survey after Q.2. 85 participants declared that they did use ultrasound at various frequencies as described in Fig. 1. Among them, few emergency doctors were trained in using ultrasound, and only 14 (17%, n = 85) had a university training in ultrasound techniques.

### Ultrasound use and disinfection

93% (78, n = 84) of ultrasound practitioners used only gel bottle for daily routine ultrasound examination, and almost 80% (68, n = 84) did not bin the bottle 24 h after it had been opened. 41% (35, n = 85) did not know if there was a protocol for ultrasound system disinfection in their department.

### Compliance rates

1. *Per question* A compliance rate of 100% (n = 51) was observed only for one question (Q.23): the question concerning the use of sterile gloves for ultrasound-guided invasive procedures.
2. *Per part and total* The total compliance rate across the questionnaire was 66.1%. Compliance rates for each part and the most significant non-compliance answers and confidence intervals are shown in Table 1.
3. *Per participant* None of the participants achieved an individual 100% compliance rate. Only 13 of them (15% 95% CI [9.1–25.9], n = 85) had a 100% compliance rate for one of the 3 parts. It was part b/ ultrasound examination on injured skin, for all of them.
4. *Sheath checking procedure* The compliance rates were very low regarding the verification of integrity of the sheath as described in Table 2.
5. The total missing data for Q.14 to Q.32 with n = 85, was 19 (1.2% of all answers). None of the questionnaire had five or more missing answers in the compliance parts and none of them was excluded.

**Table 1:** Compliance rates with 95% confidence intervals, and description of the most significant non-compliances with the guidelines

(a) Ultrasound examination on healthy skin (n = 85)	Correct answer	n answered	n correct	%
Probe disinfection before examination	YES	85	32	38
Wearing gloves during examination	NO	85	32	38
Keyboard disinfection after examination	YES	85	16	19
Compliance rate part (a): 60.4% 95% CI [56.4–64.7]				
(b) Ultrasound examination on injured skin (n = 85)	Correct answer	n answered	n correct	%
Use of a protective sterile sheath	YES	84	35	39
Use of a disposable non-sterile protection	YES	82	44	53
Compliance rate part (b): 70.9% 95% CI [66.3–76.1]				
(c) Ultrasound-guided invasive procedure *(n* = *51: Optional part)*	Correct answer	n answered	n correct	%
Probe disinfection before procedure	YES	51	30	59%
Hand hygiene gesture executed before procedure	Hand friction with hydroalcoholic solution	51	12	23%
*Other propositions: simple handwash with soap 6% (n* = *3); Surgical scrub 71% (n* = *36)*				
Type of gel container used	Sterile monodose	50	42	84%
*Other propositions: Non sterile monodose 1% (n* = *1); Non sterile gel bottle 14% (n* = *7)*				
Use of a protective sterile sheath	YES	51	47	92%
Keyboard disinfection after procedure	YES	51	9	18%
Compliance rate part (c): 69.4% 95% CI [65.1–73.6]				
Overall compliance rate (parts (a) (b) (c)): 66.1% 95% CI [62.8–69.1]

**Table 2:** Compliance rates for sheath integrity after an ultrasound-guided invasive procedure

Checking the integrity of the protective sheath (n = 51)	Correct answer	n answered	n correct	%
*By visually inspecting for:*				
A tear or a hole on the sheath	YES	51	9	18
Blood or body fluids contamination on the probe or on the dry paper used to remove gel	YES	49	12	25
*If one of these methods identifies a risk of probe contamination:*				
Existence of a specific disinfection procedure (intermediate level)?	YES	51	5	10
Probe intermediate level of disinfection: Immersion in a disinfectant solution?	YES	51	0	0

Those compliance rates were not included in the overall compliance rates of the questionnaire shown in Table 1

### Main results

In summary, this study reveals several points to be improved by awareness-raising in the emergency services of the *Hauts-de-France* region:

- Probe disinfection before examination, especially for examinations on healthy skin, which are by far the most practiced on a daily basis.
- Treatment of environmental surfaces and of the various parts of the device after use, in particular the keyboard.
- Gel conditioning, the 250 mL bottle proving unsuitable because very often contaminated and too rarely renewed due to its frequent underuse, compared to obstetric or general imaging services.
- Contact protection equipment (sheath and non-sterile protection for single use) use and glove use for standard healthy skin procedure.
- Hygienic gestures to be adopted for a sterile procedure (hand hygiene by friction with hydroalcoholic solution, use of gel in sterile monodoses) and those to avoid (hand washing with antiseptic soap, use of non-sterile monodoses or use of gel from the bottle with sterile gloves).

### Bivariate analysis

**Fig. 2:**
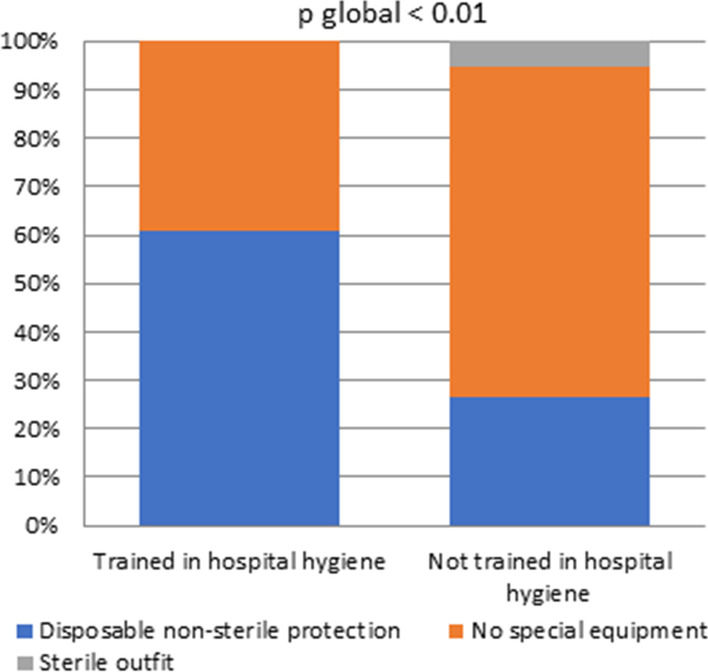
Equipment for examinations on injured skin, depending on physicians training, or not, in hospital hygiene

**Fig. 3:**
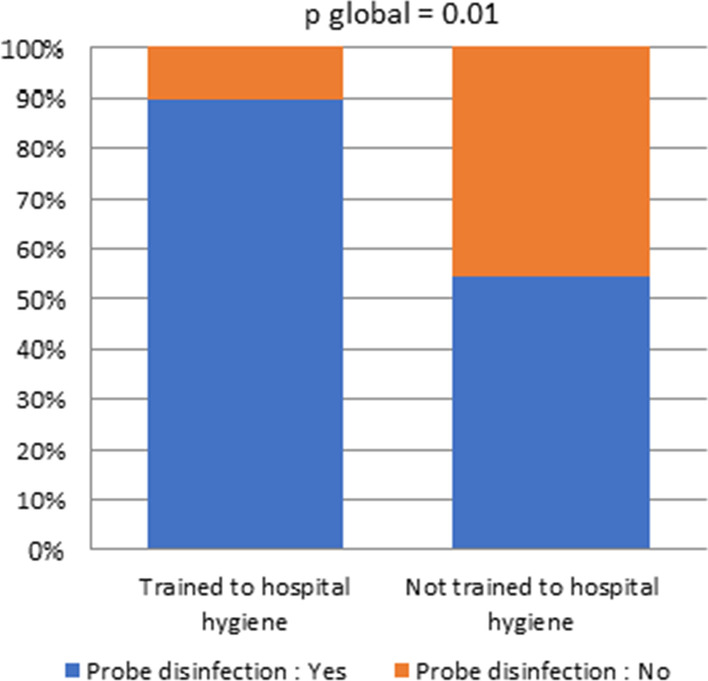
Probe disinfection after ultrasound-guided invasive procedures depending on physicians training, or not, in hospital hygiene

Bivariate analysis highlighted a statistically significant role of hospital hygiene training for 2 questions:

- The outfit used for ultrasound examination on injured skin (part (b)): only 26% of practitioners who were not trained in hygiene used a suitable gown or a disposable non-sterile protection, compared to 62% of those who were trained in hygiene. (Fig. 2, n = 85, *p* < 0.01)
- Moreover, after an ultrasound-guided invasive procedure (part (c)), 90% of the professionals trained in hospital hygiene perform a disinfection procedure (low disinfection level when no contamination has occurred) in contrast to 54% of untrained professionals (Fig. 3, n = 85, *p* = 0.01)

## Discussion

### Key results

The overall guideline compliance rate reported in this study is 66%. It is acceptable, but insufficient. Some non-compliance areas carry higher cross-contamination risks than others.

For ultrasound on healthy skin, inadequate gloves use, reported in over 60% of professionals in this study, has already been highlighted in previous audits, and has been shown to drastically impair hand hygiene [17]. Proper use of gloves thus plays a key role in adequate germ management.

For ultrasound on injured skin, although being the least common clinical situation in emergency departments, it was the only section of the survey for which some participants chose all the right answers (13 participants, so 15% of surveyed professionals, n = 85). Nonetheless, almost half of the sample (47%) reported not using personal protective equipment. 60% of them did not use the protective sheath, exposing the probe to a high risk of contact with biological fluids.

For ultrasound-guided procedures, non-compliance implies much greater risks: only one deviation from the guidelines can compromise the sterile environment. This represents a major risk if the needle used is no longer sterile at the time of puncture. Less than one out of 4 emergency doctors perform a hydro-alcoholic solution rubbing, as recommended in the guidelines. 71% of them still perform a surgical wash with iodine antiseptics, which is no longer indicated. Some emergency doctors also handle non-sterile products during the procedure: for example, 16% of them (8, n = 50), do not use a sterile gel container. Moreover, 6% of them (3, n = 51) do not use a cover at all, holding the probe with sterile gloves, and exposing it directly to body fluids. In this context, environment contamination (i.e. needle, patient, and probe) is thus unavoidable. These conclusions are in accordance with previous surveys in emergency professionals [3, 18].

For sheath integrity verification, our results are in line with European radiologists’ worrying reports of non-compliance with the existing guidelines [11]. The same guidelines were used in this study, and it is clear that emergency physicians are unaware of this hygiene procedure. Besides, none of them appear familiar with intermediate-level disinfection by soaking, as none of them reported it.

### Limitations

The form of the audit, namely a self-report, is limited due to its self-declarative nature. In addition, the questionnaire was relatively long and sometimes redundant. To reduce the potential social desirability bias, we choose to make the survey anonymous, and to not disclose that some questions were compliance questions. Indeed, this theoretically ensured that practitioners selected the behaviours they adopt themselves, instead of the ones they think they are supposed to adopt.

To further minimize this bias, we could have performed an observational audit. In fact, in addition to minimizing declaration bias, it could have allowed us to not provide detailed descriptions of the various gestures described in the guidelines. This could have enabled us to identify even more serious non-compliances, with stronger implications for patient safety.

However, we were dissuaded from proceeding this way because of the multicentric nature of the study and the impossibility of predicting routine examinations or invasive procedures for each emergency physicians willing to participate.

Another limitation of this study is that it does not offer targeted hygiene training based on its findings. The existing literature suggests that survey reminders of good practice have a significant positive impact on compliance rates [19]. Still, our bivariate analyses did not reveal enough statistically significant points to prove the benefit of hospital hygiene training, which is probably due to the low number of participants. Nonetheless, a feedback survey with detailed results and information on protocol implementation was sent to each survey coordinator.

Finally, the results were not analysed by centre due to very unequal participation rates from one centre to another. We cannot exclude a “centre effect”, as no preliminary audit on ultrasound equipment maintenance protocols has been carried out prior to this investigation.

Regarding missing data, we have estimated that their very low number did not significantly change our conclusions.

### Interpretation

The aim of this study was to determine whether emergency physicians who frequently perform ultrasound examinations are aware of the right practices to adopt in different clinical situations. The riskiest gestures, in sterile conditions, are not mastered by the participants of our study, and guideline non-compliance in routine examination situations also contributes to cross-transmission. However, in the latter, errors were less numerous and carried less risk. Still, misuse of the gel bottle, which is also an important contamination vector, carries significant risk. 93% of emergency doctors do not use a single dose of gel, and 80% of them never throw away a bottle that has been open for too long.

These findings are in line with published surveys of good practices to be observed during the repeated use of ultrasound [11]. Here, for the first time, we report similar issues in a largely understudied group of healthcare workers: emergency professionals.

Using the various recommendations from which we designed the questionnaire, we were able to highlight specific points of improvement. Consequently, we deemed it crucial to create a comprehensive protocol emphasising the most important and risk-carrying guideline non-compliances. In this protocol, the three different clinical situations studied are detailed. More specifically, for the ultrasound-guided puncture procedure, a particular emphasis is placed on the sterile nature of the material. Sheath verification after such examinations, as well as intermediate-level disinfection procedures are fully described and illustrated.

### Generalisability

The results of our study cannot be generalised to all professionals using ultrasound in a hospital context. Indeed, we have chosen to study only the population of emergency physicians using ultrasound, thereby minimizing inclusion bias. Nonetheless, the multi-centric nature of this study, its high participation rate (over 50% of centres) and the very low number of excluded questionnaires (18%, n = 19) constitute several strengths for our study. As the survey was presented as an emergency ultrasound survey by the coordinators, it was probably of specific interest to practitioners who regularly use ultrasound. We therefore believe that the results are applicable to all emergency departments. More and more emergency physicians are indeed using ultrasound routinely, but often remain a minority in their departments.

Once the disinfection protocol (designed in a simple and illustrated way) had been validated by the local scientific authorities, it was printed in the form of a laminated sheet. We then distributed it to all emergency services in the region, including hospitals that had not participated in the survey.

Furthermore, it was integrated into the ultrasound course curriculum for emergency medicine students and circulated during the presentation on infectious risk management and hygiene of reusable medical devices. It is available on the CPIAS website [20] and an English version is attached in Additional file 2.

### Guidelines evolution

In the US, the American College of Emergency Physicians has recently issued simple, concise and practical recommendations on the use and disinfection of ultrasound in the emergency ward [21]: if the level of semi-critical risk still exists, disinfection at the intermediate level is no longer mentioned, dividing the disinfection procedures into high and low levels; echo-guided gestures falling into the latter category. The possibility of sheath injury with the needle is not considered. This is of primary importance for emergency ward doctors, and it partly motivated this audit.

Besides, in 2018, the American Institute of Ultrasound in Medicine recommended the use of a sterile sheath on the probe for all intracavitary examinations or interventional ultrasound-guided procedures. In case of needle damage, a high-level disinfection must be undertaken [22].

However, these changes are not directly applied by US practitioners. In fact, this study, conducted by the World Federation in Ultrasound for Medicine and Biology also highlights problems with sheath integrity verification protocol. The conclusion highlights the need for clear guidelines, as expressed by the majority of the thousand participants [23].

In France, changes in the workplace are slower. In fact, the intermediate-level disinfection procedure, eliminated by ACEP, has just been generalised and made compulsory between each patient for examinations using EUP, under the leadership of the SF2H and its president [24]. This is an intensification of practices that were previously recommended: a single intermediary disinfection per day at the end of the program.

The main obstacle to adaptations in routine ultrasound equipment uses in the emergency room remains probe immersion. It is not certain that the ultrasound scanners in emergency departments surveyed in this study are all compatible with disinfection by soaking in a disinfectant solution. In the future, we would thus wish to bring this matter to the attention of manufacturers. Another potential solution mentioned by SF2H is the use of more effective disinfectant wipes. In fact, SF2H reports that such wipes can eliminate naked viruses and prevent Human Papilloma virus contamination of the probes between obstetric endocavitary examinations.

## Conclusion

Adoption of good hygiene practices during the repeated use of reusable medical devices can always be improved. Our study takes one example among many: the use of ultrasound by professionals working in emergency rooms. Echo-guided invasive procedures as well as simple ultrasound examinations can be performed routinely. Our work shows that aseptic and sterile environment conditions are rarely strictly respected.

In the absence of clear guidelines adapted to emergency departments and with increasing patient flows, cross contamination risks rise.

We propose a standardised protocol for ultrasound equipment disinfection based the main failures to comply with good practice recommendations revealed in our study. This protocol is ready to use and has been approved by the regional scientific authorities.

In the future, we could use an identical questionnaire or conduct an observational audit in order to define the impact of our protocol on ultrasound hygiene practices in emergency wards. It could also be useful to make more use of feedback and to provide short and local hygiene training courses for emergency department professionals.

Overall, French recommendations tend to be aligned with European and American guidelines. It is crucial to improve practitioners’ awareness of and compliance with those guidelines, to reduce cross-transmission risks.

## Supplementary Information

**Additional file 1.** Ultrasound disinfection practices protocol.

**Additional file 2.** Ultrasound equipment disinfection protocol.

## Data Availability

The data that support the findings of this study are available from the ‘Réseau Santé Qualité Risques’ of Hauts-de-France region, but restrictions apply to the availability of these data, which were used under license for the current study, and so are not publicly available. Data are however available from the authors upon reasonable request and with permission of the ‘Réseau Santé Qualité Risques’ (https://www.rsqr-hdf.com/).

## Abbreviations

APP: Assessment of professional practices
CPIAS: Centre d’appui et de prévention des infections associées aux soins (Centre for support and prevention of healthcare-associated infections)
ERS: European Radiology Society
EUP: Endocavitary ultrasound probe
SF2H: Société française d’hygiène hospitalière (French Society of Hospital Hygiene)
