# Supplementary material for: Emergency ward ultrasound: clinical audit on disinfection practices during routine and sterile examinations
Source: Antimicrob Resist Infect Control. 2021 Jan 30;10:25. doi: 10.1186/s13756-021-00896-w (PMC7847554; doi:10.1186/s13756-021-00896-w)
Supplement: Supplementary file 1 — Additional file 1. Ultrasound disinfection practices protocol. [file 13756_2021_896_MOESM1_ESM.pdf]

Questionnaire

Activity and status

Where do you work ?

☐ Public hospital ☐ Private hospital ☐ Other

What is your position ?

☐ Professor / Hospital physician, in EM ☐ Attending physician in EM ☐ Resident in EM

☐ Other (precise) :..

How old are you ?

☐ < 30 years old ☐ 30 / 45 years old ☐ > 45 years old

1/ Have you had basic training in hospital hygiene ? (hand hygiene, standard precaution…)

☐ Yes ☐ No

If yes :

☐ Less than 5 years ago ☐ More than 5 years ago

2/ How many ultrasound examinations do you perform on an average working day ?

☐ None ☐ Less than 1 per day ☐ 1 to 3 per day ☐ More than 3 per day

IF YOUR ANSWER IS « NONE », PLEASE SKIP THE FOLLOWING AND GO DIRECTLY TO QUESTION N°34

3/ Do you have a university degree in ultrasound examination ?

☐ Yes ☐ No ☐ In progress ☐ Other diploma :.................................

4/ What type of ultrasound probe do you usually use ?

Transthoracic probe ?	Abdominal probe ?	Vascular probe ?
☐ Yes ☐ No	☐ Yes ☐ No	☐ Yes ☐ No

Professional clothing

5/ For the ultrasound examination, what equipment do you wear ?

Professional outfit with short sleeves and trousers	☐ Yes ☐ No
Medical gown over your street clothes	☐ Yes ☐ No
Street clothes only	☐ Yes ☐ No
Other	☐ Yes ☐ No

6/ Generally at work, do you wear :

Rings	☐ Yes ☐ No
Watch	☐ Yes ☐ No
Nail polish	☐ Yes ☐ No

Medical device cleaning

7/ Do you use a disposable protective sheath for each ultrasound examination ?

☐ Yes ☐ No

8/ Do you have a room dedicated to ultrasound examinations, in your emergency department ?

☐ Yes ☐ No

9/ What kind of gel container do you use for ultrasound examinations ?

Sterile monodose ☐ Non-sterile monodose ☐ Gel bottle (multidose) ☐

10/ If you use gel bottles, is the bottle thrown in the bin at the end of the day ?

☐ Yes ☐ No ☐ I don't know

11/ Is there a cleaning procedure for ultrasound machines and probes ?

☐ Yes ☐ No ☐ I don't know

12/ Do you have disinfectant/detergent wipes available near the ultrasound machine ?

☐ Yes ☐ No ☐ I don't know

If yes, what is the name of the cleaning product used ?
:……………………………………………………………………………………………………

13/ Is there a record to check :

Probes maintenance ?
☐ Yes ☐ No

Device maintenance (keyboard, scroll wheel) ?
☐ Yes ☐ No

Ultrasound examinations on healthy skin

14/ If it is the first daily use of the ultrasound machine, do you clean the probe with a disinfectant/detergent wipe before starting the examination ?

☐ Yes ☐ No ☐ Sometimes

15/ Before starting the examination, do you execute hand hygiene gesture ?

☐ Yes ☐ No ☐ Sometimes

If yes :

☐ Simple hand washing ☐ Hand friction with hydroalcoholic solution

16/ Do you wear gloves during ultrasound examination ?

☐ Yes, always ☐ Yes, sometimes ☐ Never

17/ After the examination, do you clean the probe ?

☐ Yes ☐ No ☐ Sometimes

18/ After the examination, do you clean the keyboard and the scroll wheel ?

☐ Yes ☐ No ☐ Sometimes

If yes (questions 17 and 18), what is the name of the cleaning product used ?
:……………………………………………………………………………………………

19/ After examination, do you execute hand hygiene gesture ?

☐ Yes ☐ No ☐ Sometimes

If yes :

☐ Simple hand washing ☐ Hand friction with hydroalcoholic solution

Ultrasound examination on injured skin (wound, purulent skin infection, skin necrosis…)

20/ Do you wear gloves during ultrasound examination ?

☐ Yes, sterile gloves ☐ Yes, non-sterile gloves ☐ No

21/ Do you use a disposable protective sheath for the ultrasound probe ?

☐ Yes, a sterile one ☐ Yes, a non-sterile one ☐ No

22/ What kind of equipment do you wear ?

☐ Sterile outfit ☐ Disposable non-sterile protection (overcoat) ☐ No special equipment

23/ After the examination, do you clean the probe ?

☐ Yes ☐ No ☐ Sometimes

If yes, what is the name of the cleaning product used ?
:……………………………………………………………………………………………

Ultrasound-guided invasive procedures (cavity punction, central venous catheter…)

IF YOU DON'T DO / HAVE NEVER DONE ANY ULTRASOUND-GUIDED INVASIVE PROCEDURE, PLEASE SKIP THE FOLLOWING AND GO DIRECTLY TO QUESTION N°33

24/ If it is the first daily use of the ultrasound machine, do you clean the probe ?

☐ Yes, by using a disinfectant/detergent wipe

☐ Yes, by immersing the probe in a disinfectant solution, rinsing it with sterile water and drying it with sterile compress

☐ No special care

25/ Before starting the examination, do you execute hand hygiene gesture ?

☐ Simple hand washing ☐ Hand friction with hydroalcoholic solution

☐ Surgical hand washing with antiseptic solution ☐ No

26/ What kind of equipment do you wear ?

☐ Sterile outfit ☐ Disposable non-sterile protection (overcoat) ☐ No special equipment

27/ Do you wear gloves during the examination ?

☐ Yes, sterile gloves ☐ Yes, non-sterile gloves ☐ No

28/ Do you use a disposable protective sheath for the ultrasound probe ?

☐ Yes, a sterile one ☐ Yes, a non-sterile one ☐ No

29/ What kind of gel container do you use ?

☐ Sterile monodose ☐ Non-sterile monodose ☐ Gel bottle

30/ After the examination :
30-1/ Do you check the sheath visually, to look for a tear ?

☐ Yes ☐ No

30-2/ Do you wipe the probe with a dry compress once the sheath is removed, looking for contamination with fluids ?

☐ Yes ☐ No

30-3/ Do you clean the probe with a disinfectant/detergent wipe ?

☐ Yes ☐ No

31/ If you find a tear in the sheath, or contamination traces on the compress :
31-1/ Is there a specific cleaning procedure for the ultrasound probe ?

☐ Yes ☐ No ☐ I don't know

31-2/ Do you clean or ask someone to clean the probe by immersing it in a disinfectant solution bath ?

☐ Yes ☐ No ☐ Other practice : ....

32/ At the very end of the procedure, do you clean, or ask someone to clean the ultrasound machine entirely, including keyboard, scroll wheel and cables ?

☐ Yes ☐ No

Self evaluation

33/ How do you estimate your grade regarding hygiene during an ultrasound examination ?

☐ Very satisfying ☐ Satisfying ☐ Unsatisfactory ☐ Very unsatisfactory

34/ How do you estimate the need of general hygiene training for emergency physicans, in Nord-Pas-de-Calais ?

☐ Very necessary ☐ Necessary ☐ Incidental ☐ Useless

35/ Would you be interested if the 'Collège de Médecine d'Urgence' (COMU 59-62) proposed a training about infectious risk prevention ?

☐ Very interested ☐ Interested ☐ Moderalty interested ☐ Not interested
