# Supplementary material for: Emergency ward ultrasound: clinical audit on disinfection practices during routine and sterile examinations
Source: Antimicrob Resist Infect Control. 2021 Jan 30;10:25. doi: 10.1186/s13756-021-00896-w (PMC7847554; doi:10.1186/s13756-021-00896-w)
Supplement: Supplementary file 2 — Additional file 2. Ultrasound equipment disinfection protocol. [file 13756_2021_896_MOESM2_ESM.pdf]

ULTRASOUND EQUIPMENT DISINFECTION PROTOCOL

According to the actual recommendations[1]

Collège de Médecine d'Urgence Nord-Pas-de-Calais

Taken from the work of Axel Andolfo,
emergency physican in 'Centre Hospitalier de Dunkerque',
With the support of 'COMU59-62', 'CPIAS Hauts-de-France' and 'Réseau Santé Qualité'

CPias Hauts-de-France

1/ Common gestures to all kinds of examinations

BEFORE AND AFTER EXAMINATION

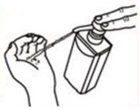

Hand friction (HF) with hydroalcoholic solution

BEFORE US EXAM.

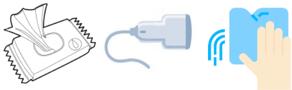

Probe disinfection with a detergent/disinfectant wipe

AFTER US EXAM.

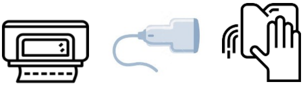

Clean the probe by removing the gel with a dry paper on

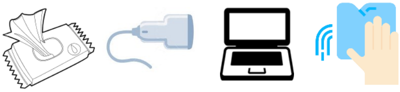

Ultrasound equipment disinfection :
Cables, keyboard, scroll wheel, monitor and probe,
With a detergent/disinfectant wipe

If you have any questions concerning disinfection of Reusable Medical Devices, please call your local hygiene department.

HYGIENE TEAM

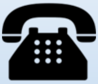

__ __ __ __

Common sequence to all kinds of examinations :

BEFORE

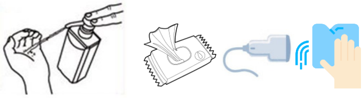

AFTER

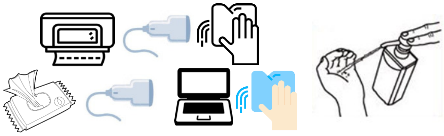

2/ Ultrasound gel

STERILE MONODOSE
For US-guided invasive procedures (sterile)

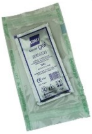

NON-STERILE MONODOSE : Is preferred
- IN ALL OTHER SITUATIONS (But sterile)
- MUST BE ALWAYS AVAILABLE

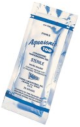

GEL BOTTLES : HAS TO BE BINNED IF OPEN FOR MORE THAN 24 HOURS

Ultrasound gel facilitates bacterial growth, just like agar in the laboratory.
All gel bottles should be considered contaminated, sometimes even before they have been opened.
They do not belong in a sterile environment.

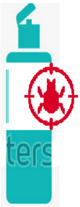

[1] SF2H, 2009 « Précautions complémentaires contact », 2018 « Hygiène des mains » // SFR, 2009 « Echographie » // SFAR, 2011 « ALR »

3/ Clinical situations

Ultrasound examination on healthy skin

BEFORE/AFTER : Common sequence
To all kind of examinations

DURING EXAMINATION : WEARING GLOVES IS NOT RECOMMENDED

Ultrasound examination on injured skin (wound, necrosis, purulent infection)

Or for patients placed on « Contact Precautions (CP) »

BEFORE/AFTER : Common sequence to all kind of examinations

DURING EXAMINATION :

- DIPOSABLE NON-STERILE PROTECTION
- **STERILE PROTECTIVE SHEATH**
- NON-STERILE GLOVES **only if there is a risk of exposure to body fluids**

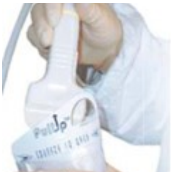

*Gloves are **not recommended** for a patient placed on CP without any risk of exposure to body fluids*

Ultrasound-guided invasive procedures

BEFORE/AFTER : Common sequence to all kind of examinations

Do a surgical hand friction by using **hydroalcoholic solution**
Use **sterile** outfit, **sterile** gloves and **sterile** protective sheath
Use gel **STERILE** monodose only

At the end of the procedure, look for probe contamination, by visually checking :

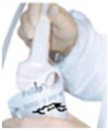

The sheath :
Looking for
tears, holes...

The probe :
Looking for
contamination with

Finally, use a dry paper to remove the gel from the probe and look for blood or any body fluids on the paper.

Collège de Médecine d'Urgence Nord-Pas-de-Calais

CPias Hauts-de-France

If you find a tear or a hole on the sheath, or if the probe has been contaminated with blood or any body fluids :

PROBE DISINFECTION BY IMMERSION IN A DISINFECTANT SOLUTION IS RECOMMENDED ***Please contact the hygiene department for further questions***
